# Further characterization of ADAMTS-13 inactivation by thrombin

**DOI:** 10.1111/j.1538-7836.2007.02514.x

**Published:** 2007-05

**Authors:** J K LAM, C K N K CHION, S ZANARDELLI, D A LANE, J T B CRAWLEY

**Affiliations:** Department of Haematology, Imperial College London London, UK

**Keywords:** ADAMTS-13, thrombin, von Willebrand factor

## Abstract

*Background:* The multimeric size and platelet-tethering function of von Willebrand factor (VWF) are modulated by the plasma metalloprotease, a disintegrin and metalloproteinase with a thrombospondin type 1 motif, member 13 (ADAMTS-13). *In vitro* ADAMTS-13 is susceptible to proteolytic inactivation by thrombin. *Objectives:* In this study, we aimed to characterize the inactivation of ADAMTS-13 by thrombin and to assess its physiological significance. *Methods and results:* By N-terminal sequencing of cleavage products, and by mutagenesis, we identified the principal thrombin cleavage sites in ADAMTS-13 as R257 and R1176. Using a library of 76 thrombin mutants, we highlighted the functional importance of exosite I on thrombin in the proteolysis of ADAMTS-13. Proteolysis of ADAMTS-13 by thrombin caused an 8-fold reduction in its affinity for VWF that contributed to its loss of VWF-cleaving function. Intriguingly, thrombin-cleaved ADAMTS-13 both bound and proteolyzed a short recombinant VWF A2 domain substrate (VWF115) normally. Following activation of coagulation in normal plasma, endogenous ADAMTS-13, but not added ADAMTS-13, appeared resistant to coagulation-induced fragmentation. An estimation of the *K*_m_ for ADAMTS-13 proteolysis by thrombin was appreciably higher than the physiological concentration of ADAMTS-13. This was corroborated by the comparatively low affinity of ADAMTS-13 for thrombin (*K*_D_ 95 nm). *Conclusions:* Together, our data suggest that ADAMTS-13 is protected from rapid proteolytic inactivation by thrombin in normal plasma. Whether this remains the case under pathological situations involving elevated/sustained generation of thrombin remains unclear.

## Introduction

von Willebrand factor (VWF) is a large, multimeric plasma glycoprotein that is expressed by endothelial cells and megakaryocytes [1]. Multimers arise through C- and N-terminal disulfide-bond formation [2]. This process generates ‘ultra-large’ multimers (UL-VWF) that can exceed 2 × 10^4^ kDa. Endothelial VWF is constitutively secreted, but also stored within Weibel–Palade bodies, predominantly as UL-VWF [3]. This pool is released upon endothelial cell activation [4]. A wide range of VWF species is found in normal plasma, differing only in the number of component VWF units.

VWF mediates rapid adhesion of platelets at sites of vascular injury. This occurs by the specific bridging of circulating platelets and the injured vessel wall. The platelet-tethering properties of VWF are dependent upon its multimeric size [5]. Larger multimers bind circulating platelets tighter, and also more readily undergo conformational changes in response to the rheological forces [6]. VWF multimers circulate in a globular conformation that does not interact with platelets. However, following vessel injury, globular VWF binds rapidly and tightly to the exposed subendothelial collagen. The increased shear forces exerted by the circulating blood cause the bound globular VWF molecule to unravel [7]. This exposes binding sites for glycoprotein Ibα (a receptor present on the surface of circulating platelets) in the VWF A1 domain that leads to the local recruitment of platelets, and the formation of a primary platelet plug [8].

VWF multimeric size/platelet-tethering function is modulated by the plasma metalloprotease, a disintegrin and metalloproteinase with a thrombospondin type 1 motif, member 13 (ADAMTS-13), which cleaves VWF at a single site in the A2 domain [9,10]. This converts UL-VWF secreted by endothelial cells into lower-molecular-weight forms with reduced adhesive potential. ADAMTS-13 is a ∼190 kDa multidomain plasma glycoprotein [11]. The metalloprotease domain at the N-terminus of ADAMTS-13 contains a highly conserved active site responsible for VWF A2 domain proteolysis. The disintegrin-like domain and the first thrombospondin-1 repeat domain (TSP) precede the Cys-rich domain. The Spacer domain is essential for full ADAMTS-13 activity and binds to the C-terminal aspect of the VWF A2 domain [12,13]. This domain links the Cys-rich region to seven consecutive TSP repeats. Unlike other ADAMTS family members, ADAMTS-13 has two C-terminal CUB domains. The first CUB domain may be involved in modulating ADAMTS-13 activity, or in helping to attach ADAMTS-13 to the A3 domain of UL-VWF strings under flowing conditions [14,15]. With the exception of the metalloprotease and Spacer domains, the precise role that each ADAMTS-13 domain plays remains unclear. ADAMTS-13 is expressed predominantly by the liver and secreted into the blood as an active enzyme (plasma concentration of ∼5 nm) [16]. ADAMTS-13 is also expressed by endothelial cells in culture [17,18].

How ADAMTS-13 activity is regulated has yet to be defined. We previously reported that ADAMTS-13 is susceptible to proteolytic fragmentation by thrombin [and also factor (F) Xa and plasmin], and that this ablates its VWF-cleaving function [19]. Proteolysis might represent a mechanism by which ADAMTS-13 function is locally modulated at sites of vascular injury, thereby limiting VWF proteolysis and reducing platelet embolization. To better understand both the mode and the physiological significance of this process, we have characterized further the proteolysis and inactivation of ADAMTS-13 by thrombin.

## Materials and methods

### Expression and purification of ADAMTS-13

Wild-type (WT) recombinant ADAMTS-13 (rADAMTS-13) with a C-terminal myc/His tag was expressed in HEK293 cells and purified as previously described [19,20]. R257A and R1176H ADAMTS-13 mutants were generated using Quikchange site-directed mutagenesis, according to manufacturer's instructions (Stratagene, Amsterdam, The Netherlands), and verified by sequencing. For transient transfection and expression, the pcDNA3.1/ADAMTS-13-myc/His expression vector (or R257A and R1176H mutants) was transfected into 10 cm dishes of 70% confluent HEK293T cells using Lipofectamine (Invitrogen, Paisley, UK), according to manufacturer's instructions. Conditioned media were collected 3 days post-transfection. Cell debris was removed and samples dialyzed into 20 mm Tris (pH 7.8). ADAMTS-13 concentrations were determined using a specific ELISA [21].

### Proteolysis of ADAMTS-13 by thrombin

To assess proteolysis *in vitro*, reactions containing rADAMTS-13 (or mutant), thrombin (Sigma, Poole, UK), 150 mm NaCl, 5 mm CaCl_2_, 20 mm Tris (pH 7.8) were incubated at 37 °C. Subsamples were removed at designated time-points, and stopped with EDTA. The amount of thrombin and ADAMTS-13 used varied according to the experiment and is given for each experiment in the Results section and in the figure legends. Proteolysis of ADAMTS-13 was assessed by Western blotting under reducing conditions using either an antimetalloprotease domain monoclonal antibody (mAb), an anti-CUB domain mAb (both generously provided by Dr F. Scheiflinger, Baxter, Vienna, Austria), or an anti-ADAMTS-13 polyclonal antibody (pAb) generated and purified in our laboratory [21]. To assess the contribution of surface residues on thrombin in the proteolysis of ADAMTS-13, a library of 76 single surface residue thrombin point mutants was used (kindly provided by Dr L. Leung, Stanford University, CA, USA) [22]. All mutants were active-site titrated using S-2238, as previously described [22], to ensure that the same specific activity was used. WT thrombin (or mutant) was added to each reaction, containing 250 nm ADAMTS-13, and incubated for 0–2 h. Western blotting was then performed using both the anti-CUB domain and antimetalloprotease domain mAbs.

In reactions containing VWF or fibrinogen, 50 nm ADAMTS-13 was preincubated at 37 °C for 20 min with or without 500 nm plasma-derived VWF (gel purified from Haemate P, and quantified as described previously [23]), or 7 μm fibrinogen (Quadratech, Surrey, UK). Thereafter, 100 nm thrombin was added and incubated as above. Proteolysis was assessed using the anti-ADAMTS-13 pAb or antimetalloprotease domain mAb.

For N-terminal sequencing, 1 μm ADAMTS-13 was incubated with 100 nm thrombin for 6 h and subjected to sodium dodecylsulfate polyacrylamide gel electrophoresis (SDS–PAGE) on 16% acrylamide Tris-Tricine gels under reducing conditions. Bands were excised and subjected to N-terminal sequencing (Alta Bioscience, Birmingham, UK). Potential thrombin cleavage sites were identified *in silico* using PeptideCutter software (http://us.expasy.org/tools/peptidecutter/).

### Proteolysis of ADAMTS-13 in plasma

In plasma experiments, coagulation was initiated in citrated normal human plasma, or plasma from afibrinogenemic patients (George King Biomedical, Overland Park, KS, USA) by the addition of 8 pm lipidated tissue factor (Baxter), Ca^2+^, and 4 μm phospholipids (phosphatidylserine:phosphatidylcholine:phosphatidylethanolamine – 20:60:20; Avanti, Alabaster, AL, USA), as previously described [19]. To prevent clot formation and to allow Western blot analysis, 5 mg mL^−1^ Gly-Pro-Arg-Pro-amide (Sigma), which allows fibrinogen cleavage but prevents fibrin polymerisation, was included. After 30 min, samples were analyzed by Western blotting using the anti-ADAMTS-13 pAb. Thrombin generation was monitored in parallel samples, as previously described [19].

### ADAMTS-13 activity assays

Two ADAMTS-13 activity assays were used [with either plasma-derived multimeric VWF or a recombinant bacterially expressed A2 domain fragment (VWF115) as a substrate] [19,20]. For competition experiments, ADAMTS-13 was preincubated with or without 100 nm thrombin for 6 h at 37 °C. A molar excess of hirudin (Calbiochem, Nottingham, UK) was added to specifically inhibit thrombin [19]. Activity assays containing either 6 nm ADAMTS-13, 60 nm thrombin-cleaved ADAMTS-13, or a mixture containing 6 nm ADAMTS-13 and 60 nm thrombin-cleaved ADAMTS-13 were incubated at 37 °C with 10 nm VWF, 5 mm BaCl_2_, 1.5 m urea, 50 mm NaCl, 20 mm Tris (pH 7.8). Subsamples were removed from 0 to 4 h, and stopped with EDTA. The proteolysis of VWF by ADAMTS-13 was assessed by measuring the loss of collagen binding function, as previously described [19,23].

For the VWF115 assay, 5 nm ADAMTS-13, or thrombin-cleaved ADAMTS-13, was incubated at 37 °C in reactions containing 5 μm VWF115, 150 mm NaCl, 5 mm CaCl_2_, 20 mm Tris (pH7.8). Subsamples were taken from 0 to 2 h and stopped with EDTA. Proteolysis of VWF115 was assessed qualitatively by SDS–PAGE on 16% acrylamide Tris-Tricine gels and Coomassie staining. For kinetic analysis, reactions were set up as above using 700 nm VWF115. Reactions were stopped, and VWF115 proteolysis measured by high-performance liquid chromatography. The catalytic efficiency of proteolysis was determined as previously described [20].

### ADAMTS-13 binding assays

To measure ADAMTS-13, or thrombin-cleaved ADAMTS-13 binding to multimeric VWF, VWF115 or thrombin, 30 nm VWF, 140 nm VWF115 or 80 nm active site-blocked thrombin was immobilized to the bottom of 96-well plates with 50 mm sodium carbonate (pH9.6) overnight at 4 °C. Wells were washed with phosphate-buffered saline (PBS)/0.1% Tween-20, and then blocked with 1% bovine serum albumin/PBS for 1 h. One hundred microliter 0–1 μm ADAMTS-13 (or thrombin-cleaved ADAMTS-13) was added to wells in triplicate and incubated for 2 h. Wells were washed three times with PBS/0.1% Tween-20 and bound ADAMTS-13 specifically detected with 0.5 μg mL^−1^ biotinylated anti-ADAMTS-13 pAb in blocking buffer, and in turn detected with a streptavidin–horseradish peroxidase conjugate (GE Healthcare, Buckinghamshire, UK). Bound conjugate was quantified by incubation with *o*-phenylenediamine (Sigma) and then measuring the absorbance at 492 nm.

## Results

### Identification of thrombin cleavage sites in ADAMTS-13

**Fig. 1:**
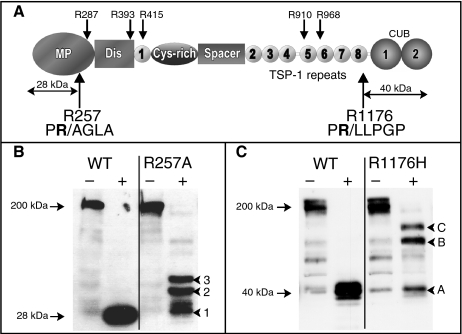
Identification of thrombin cleavage sites in ADAMTS-13. (A) Diagram of ADAMTS-13 domain structure. Locations of predicted thrombin cleavage sites after R287, R393, R415, R910 and R968 are shown above. Thrombin cleavage sites determined by N-terminal sequencing of cleavage fragments are shown below with the amino acid sequence of the amino acids that flank these sites. Single-letter amino acid code is shown, P1 arginine is highlighted in bold, cleavage site is denoted by (/). The location of the 28 and 40 kDa fragments detected in (B) and (C) are highlighted. (B) Wild-type (WT) ADAMTS-13 and ADAMTS-13(R257A) mutant were incubated with and without (+ and –) 10 nm thrombin for 6 h and analyzed by Western blotting using an antimetalloprotease domain monoclonal antibody (mAb). Proteolysis after R257 was prevented by substitution of the P1 arginine to alanine, and resulted in detection of alternative cleavage fragments 1, 2 and 3. (C) WT ADAMTS-13 and ADAMTS-13(R1176H) mutant were incubated with and without (+ and –) thrombin as in (B), and analyzed by Western blotting using an anti-CUB mAb. Proteolysis after R1176 was significantly inhibited by substitution of the P1 arginine to histidine resulting in detection of cleavage fragments A, B and C.

ADAMTS-13 is rapidly proteolyzed and inactivated by thrombin in a purified system [19]. To characterize this inactivation, we aimed to delineate the precise sites in ADAMTS-13 at which thrombin cleaves. There are at least six thrombin cleavage sites in ADAMTS-13. Of these, two are proteolyzed preferentially: one site located close to the carboxyl end of the metalloprotease domain, and the other in between the TSP8 domain and the CUB domains [19]. Analysis of the primary amino acid sequence of ADAMTS-13 using PeptideCutter software revealed the location of seven potential thrombin cleavage sites: after R257 and R287 (metalloprotease domain), R393 and R415 (TSP1), R910 (TSP5), R968 (TSP6), and R1176 (in between TSP8 and CUB1) (Fig. 1A). Despite these predictions, it remained unclear whether these were the actual sites of proteolysis. We therefore separated ADAMTS-13 proteolytic fragments by SDS–PAGE under reducing conditions, as all proteolytic fragments remain covalently associated by disulfide bonds with the exception of the C-terminal CUB domain-containing fragment [19]. Bands of 28, 24 and 40 kDa were resolved and their N-termini determined by Edman degradation. The 28 kDa fragment had an N-terminal sequence of GILHLE, corresponding to the mature ADAMTS-13 N-terminus (i.e. the start of the metalloprotease domain). The 24 kDa fragment had the sequence AGLA, representing a fragment generated by proteolysis after R257 (Fig. 1A). This demonstrated that R257 represents the favored most N-terminal cleavage site. The N-terminal sequence of the 40 kDa fragment was LLPGP. This corresponds to the most C-terminal cleavage site, and arose through proteolysis after R1176 (Fig. 1A). Other proteolytic fragments could not be adequately resolved on the gel - either from each other or from thrombin – in order to enable Edman degradation.

To further corroborate these findings we mutated the predicted P1 (thrombin cleavage site) arginine residues in ADAMTS-13 to prevent proteolysis at these sites. The region surrounding R257 exhibits no homology amongst ADAMTS family members, suggesting that this residue does not fulfill a conserved structural role. Consequently, R257 was mutated to alanine. R1176 was mutated to histidine to preserve the basic nature of this amino acid. Both mutants were expressed/secreted normally, and exhibited normal activity (data not shown), suggesting that the substitutions did not significantly influence either the structure or the function of ADAMTS-13.

Incubation of these mutants with thrombin confirmed R257 and R1176 as the two favored P1 cleavage site residues. After proteolysis of WT ADAMTS-13 with 10 nm thrombin for 6 h, no full-length ADAMTS-13 was detected by Western blotting using an antimetalloprotease domain mAb. Instead, only a single 28 kDa proteolytic fragment was observed. However, whereas similar treatment of ADAMTS-13(R257A) demonstrated a comparable loss of full-length ADAMTS-13, a mixture of metalloprotease domain-containing fragments with molecular masses greater than 28 kDa were detected (fragments 1, 2 and 3: ∼30, ∼40 and ∼45 kDa, respectively), indicating that proteolysis after R257 had been prevented (Fig. 1B). Fragments 1, 2 and 3 possibly represent cleavage products arising from proteolysis after the predicted/less-favored R287, R393 and R415 sites, respectively. The detection of a mixture of fragments also confirmed that proteolysis after R257 occurs more rapidly than at these other sites.

Analogous analysis of ADAMTS-13(R1176H) was performed except that fragmentation was analyzed with an anti-CUB mAb in order to more accurately monitor proteolysis at this site. Similar to before, thrombin treatment of ADAMTS-13 resulted in complete disappearance of full-length ADAMTS-13 when detected with this mAb (Fig. 1C). The only cleavage product detected was the 40 kDa CUB domain-containing fragment. Proteolysis of ADAMTS-13(R1176H) revealed a mixture of cleavage fragments (fragments A, B and C: ∼40, ∼70 and ∼80 kDa, respectively). Although thrombin specifically cleaves after certain arginine (or lysine) residues, it is also capable of doing so after histidine, albeit at an appreciably reduced (approximately one hundred sixtyfold) rate [24]. Fragment A most likely represents the product of proteolysis after the R1176H substitution. Fragments B and C are potentially fragments arising from cleavage after R968 and R910, respectively. The lack of information about the sites/extent of glycosylation in ADAMTS-13 makes it difficult to accurately predict the sites of proteolysis that generate fragments 1, 2, 3, B and C based on their molecular mass.

### Identification of thrombin exosite I residues important for proteolysis of ADAMTS-13

**Fig. 2:**
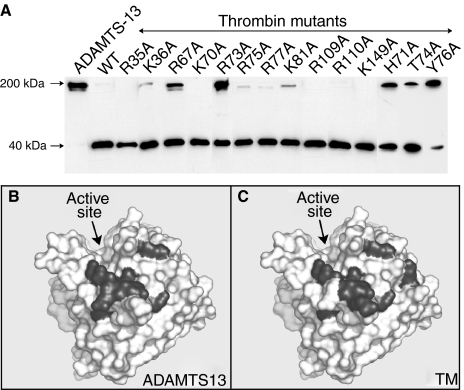
Proteolysis of ADAMTS-13 by thrombin mutants. (A) ADAMTS-13 was incubated with 100 nm of wild-type (WT) thrombin or thrombin mutants carrying substitutions in surface-exposed residues. After 2 h, ADAMTS-13 fragmentation by each different thrombin variant was assessed by Western blotting using an anti-CUB domain monoclonal antibody. By comparison with WT thrombin the variants with substitutions at K36, R67, H71, R73, T74, R75, Y76, R77 and K81 (chymotrypsin numbering) exhibited reduced ability to cleave ADAMTS-13. Space-fill model of thrombin is shown. The active site cleft is highlighted. Those residues shown in grey were determined to be important for proteolysis of ADAMTS-13 (B) and were all located in exosite I of thrombin. The location of these amino acids is very similar to those previously demonstrated to be important for binding thrombomodulin (C).

To determine how thrombin interacts with ADAMTS-13, we used a library of 76 thrombin variants that contained single (predominantly charged) residue substitutions to alanine of surface-exposed amino acids [22]. Proteolysis of ADAMTS-13 by WT thrombin, or thrombin mutants, was assessed by Western blot analysis using the anti-CUB mAb. Under these conditions, the complete disappearance of full-length ADAMTS-13 occurred after 2 h with WT thrombin. Of all the thrombin mutants tested, only those with substitutions located in exosite I of thrombin exhibited a reduced propensity to cleave ADAMTS-13, as seen by the presence of uncleaved ADAMTS-13 after 2-h incubation with these variants (Fig. 2A; not all variants tested are shown). Results were confirmed by analysis using the antimetalloprotease domain mAb (not shown). The thrombin variants with reduced ADAMTS-13-cleaving function contained substitutions at K36, R67, H71, R73, T74, R75, Y76, R77 and K81 (chymotrypsin numbering). These residues are broadly similar to the exosite I residues in thrombin that are important in the binding of thrombomodulin (TM; Fig. 2B,C) [22].

### ADAMTS-13 proteolyzed/inactivated by thrombin binds VWF, but with reduced affinity

**Fig. 3:**
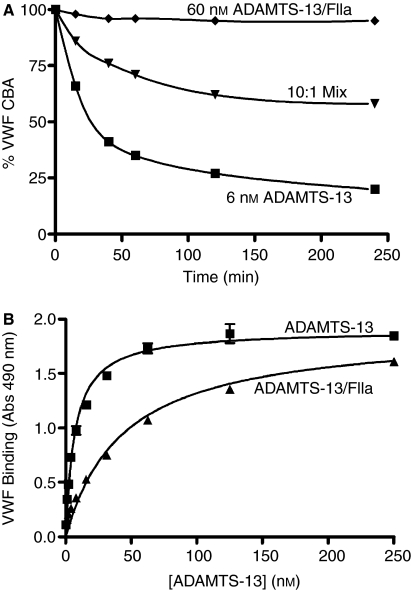
ADAMTS-13 proteolyzed by thrombin has reduced affinity for von Willebrand factor (VWF). (A) 6 nm ADAMTS-13, 60 nm thrombin-cleaved ADAMTS-13 (ADAMTS-13/FIIa) or 60 nm thrombin-cleaved ADAMTS-13/6 nm ADAMTS-13 (10:1 mix) was incubated with 10 nm purified plasma-derived VWF in the presence of 1.5 m urea and 5 mm BaCl_2_. At time-points (0–4 h) subsamples were stopped with EDTA and VWF function measured using a collagen-binding assay (CBA). Changes in CBA are represented as % original CBA at 0 h. A 10-fold molar excess of proteolyzed ADAMTS-13 partially competed with ADAMTS-13 for VWF. (B) The affinity of ADAMTS-13 and thrombin-cleaved ADAMTS-13 for purified plasma-derived VWF was measured by plate assay. 30 nm VWF was immobilized to microtiter wells, and incubated with varying concentrations of ADAMTS-13 or thrombin-cleaved ADAMTS-13 (ADAMTS-13/FIIa), as in Materials and methods. A high-affinity interaction between ADAMTS-13 and VWF was determined (K_D_∼6 nm), whereas this was appreciably reduced for ADAMTS-13/FIIa (*K*_D_∼45 nm).

Although proteolysis of ADAMTS-13 by thrombin prevents its ability to cleave VWF, how this inactivation occurs was uncertain. To examine this, the ability of thrombin-cleaved ADAMTS-13 to compete with ADAMTS-13 for VWF was tested. As expected, ADAMTS-13 (6 nm) reduced the collagen-binding function of VWF over time, whereas a 10-fold increased amount of thrombin-cleaved ADAMTS-13 (60 nm) was unable to do so (Fig. 3A). However, when 6 nm ADAMTS-13 was mixed with 60 nm thrombin-treated ADAMTS-13 (containing an excess of hirudin to inhibit thrombin), the rate of loss of collagen-binding function was reduced when compared with 6 nm ADAMTS-13 alone. This demonstrated that proteolyzed ADAMTS-13 could block the proteolysis of VWF by uncleaved ADAMTS-13, and suggests that ADAMTS-13 retains some VWF-binding function after thrombin cleavage.

The binding of thrombin-cleaved ADAMTS-13 to VWF was analyzed using an equilibrium plate binding assay (Fig. 3B). ADAMTS-13 bound immobilized VWF with high affinity (*K*_D_ 6 nm), similar to previous estimates [25]. Although proteolyzed ADAMTS-13 bound VWF with the same capacity, it did so with approximately eightfold reduced affinity (*K*_D_ 45 nm).

### ADAMTS-13 proteolyzed by thrombin remains fully activated against VWF115

**Fig. 4:**
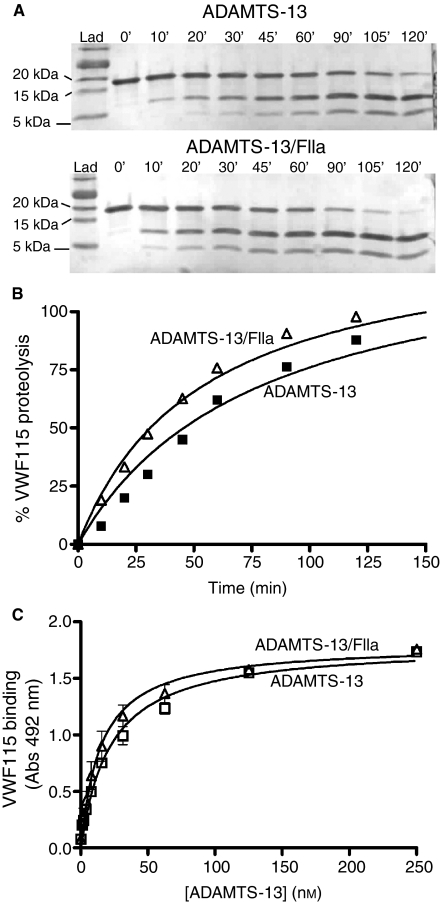
Proteolysis of ADAMTS-13 by thrombin does not influence the cleavage of, or binding to, VWF115. (A) 5 nm ADAMTS-13 or thrombin-cleaved ADAMTS-13 (ADAMTS-13/FIIa) was incubated at 37 °C with 5 μm VWF115, 150 mm NaCl, 5 mm CaCl_2_, 20 mm Tris (pH7.8). Subsamples were stopped with EDTA (0–2 h) and analyzed by sodium dodecylsulfate polyacrylamide gel electrophoresis and Coomassie staining. Proteolysis of VWF115 was visualized by the disappearance of a 16.9 kDa band and the appearance of 10 kDa and 6.9 kDa cleavage products. (B) Kinetic analysis of VWF115 proteolysis, as in (A) except using 700 nm VWF115. Proteolysis of VWF115 was quantified by high-performance liquid chromatography, and represented graphically. The catalytic efficiency for VWF115 proteolysis was derived from fitted data for both ADAMTS-13 and ADAMTS-13/FIIa (8 × 10^4^ m^−1^ s^−1^ and 11 × 10^4^ m^−1^ s^−1^, respectively). (C) The affinity of ADAMTS-13 and thrombin-cleaved ADAMTS-13 for VWF115 was measured by plate assay. 140 nm VWF115 was immobilized to microtiter wells, and incubated with varying concentrations of ADAMTS-13 or thrombin-cleaved ADAMTS-13 (ADAMTS-13/FIIa), as in Materials and methods. A similar high-affinity interaction was determined (*K*_D_∼20 nm) for both ADAMTS-13 and ADAMTS-13/FIIa with VWF115.

To examine the inactivation of ADAMTS-13 by thrombin kinetically, we employed a previously characterized assay using a recombinant A2 domain fragment, VWF115 [20]. Substrate proteolysis was first visualized by SDS–PAGE and Coomassie staining (Fig. 4A). Surprisingly, thrombin-cleaved ADAMTS-13 generated VWF115 cleavage products at a similar rate to ADAMTS-13. However, a major difference between the VWF115 and the multimeric VWF activity assays is the presence of urea. To ensure that it was not the denaturant that inactivated proteolyzed ADAMTS-13, we examined VWF115 proteolysis in the presence and absence of 1.5 m urea. These data demonstrated that the ability of thrombin-cleaved ADAMTS-13 to proteolyze VWF115 was not influenced by these mild denaturing conditions (data not shown). Kinetic analysis of VWF115 cleavage revealed that the catalytic efficiency of cleavage (*k*_cat_/*K*_m_) for both ADAMTS-13 and thrombin-cleaved ADAMTS-13 (8 × 10^4^ m^−1^ s^−1^ and 11 × 10^4^ m^−1^ s^−1^, respectively) were very similar to previous data derived for ADAMTS-13 [20] (Fig. 4B). A binding assay using immobilized VWF115 corroborated these results by demonstrating that both ADAMTS-13 and thrombin-cleaved ADAMTS-13 bound VWF115 with equally high affinity (*K*_D_∼18 nm) (Fig. 4C). Together, these data demonstrate that whereas thrombin proteolysis inhibits the ability of ADAMTS-13 to cleave multimeric VWF, it has no effect upon its ability to cleave short substrates. Thus, despite the presence of a cleavage site within the ADAMTS-13 metalloprotease domain, rather surprisingly it appears that this domain remains fully functional after proteolysis by thrombin.

### Proteolysis of endogenous ADAMTS-13 by thrombin does not occur in plasma

**Fig. 5:**
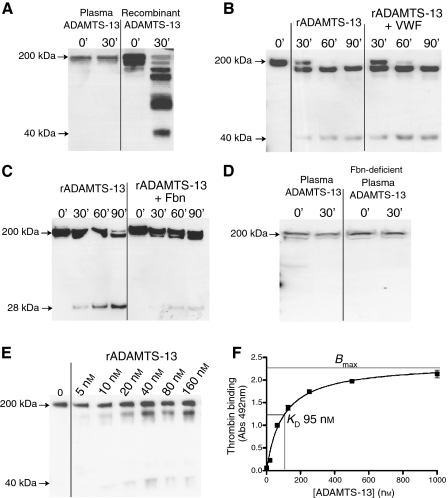
ADAMTS-13 is not efficiently proteolyzed by thrombin in plasma. (A) Endogenous ADAMTS-13 was detected in plasma by Western blotting using an anti-ADAMTS-13 polyclonal antibody (pAb). No change in the intensity of this band was observed 30 min after initiation of coagulation. Conversely, multiple cleavage products were generated when 100 nm recombinant ADAMTS-13 (rADAMTS-13) was incubated with 100 nm thrombin for 30 min and analyzed in the same way. (B) 50 nm rADAMTS-13 was incubated with 100 nm thrombin in the presence and absence of 500 nm von Willebrand factor. ADAMTS-13 fragmentation was analyzed from 0–90 min by Western blotting with an anti-ADAMTS-13 pAb. (C) 100 nm rADAMTS-13 was incubated with thrombin in the presence and absence of 7 μm human fibrinogen (Fbn). ADAMTS-13 fragmentation was analyzed from 0–90 min by Western blotting with an antimetalloprotease domain monoclonal antibody. (D) Endogenous plasma ADAMTS-13 was detected in normal plasma and plasma from afibrinogenemic patients (Fbn-deficient) as in (A). No change in the intensity of the ADAMTS-13 band was observed 30 min after initiation of coagulation in either plasma. (E) 5–160 nm rADAMTS-13 was incubated with 25 nm thrombin for 60 min and thereafter ADAMTS-13 fragmentation was analyzed as in (A). ADAMTS-13 prior to thrombin incubation (0′) is shown. For each concentration, the same amount of ADAMTS-13 was used for Western blotting. (F) The affinity of ADAMTS-13 for thrombin was measured by plate assay. 80 nm active site blocked thrombin was immobilized to microtiter wells, and incubated with varying concentrations of rADAMTS-13, as in Materials and methods. A *K*_D_∼95 nm was determined.

In an earlier study, we examined the proteolysis of ADAMTS-13 following activation of coagulation in plasma [19]. However, the relatively low affinity of the available antibodies did not allow detection of endogenous ADAMTS-13 in plasma. Consequently, we were obliged to supplement normal plasma with rADAMTS-13 in order to enable its detection. Using more sensitive anti-ADAMTS-13 pAb, we now examined the influence of coagulation upon endogenous ADAMTS-13. Following initiation of coagulation in normal human plasma, we detected no change in the intensity of the full-length endogenous ADAMTS-13 band after 30 min (Fig. 5A). This suggested that whereas rADAMTS-13 is efficiently cleaved by thrombin *in vitro*, endogenous plasma ADAMTS-13 is in some way protected from this proteolysis.

To rationalize this observation, we postulated that at normal physiological ADAMTS-13 concentrations (∼5 nm), other components of plasma might influence its proteolysis. First, we hypothesized that the binding of VWF to ADAMTS-13 might protect it from thrombin. To test this we preincubated ADAMTS-13 with and without a 10-fold molar excess of purified multimeric VWF (approximate physiological ratio) prior to addition of thrombin. Analysis by Western blotting revealed no difference in the rate of ADAMTS-13 cleavage in the presence/absence of VWF (Fig. 5B), indicating that once bound to VWF, ADAMTS-13 remains equally susceptible to thrombin cleavage *in vitro*.

We next examined whether other thrombin substrates might impair the ability of thrombin to proteolyze ADAMTS-13 in plasma. Fibrinogen is the most abundant thrombin substrate in plasma. Incubation of ADAMTS-13 with thrombin in both the presence and absence of 7 μm fibrinogen revealed that appreciable, if not complete, competition occurred. Fibrinogen reduced the rate at which ADAMTS-13 was proteolyzed by thrombin (Fig. 5C), consistent with their similar dependence upon exosite I of thrombin (Fig. 2). To ascertain whether this accounted for the lack of proteolysis of endogenous ADAMTS-13 in plasma, we initiated coagulation in plasma from afibrinogenemic patients (containing less than 1% normal fibrinogen levels), and examined ADAMTS-13 proteolysis as before (Fig. 5D). However, despite the near absence of fibrinogen, the intensity of the endogenous full-length ADAMTS-13 band remained unaltered after 30 min.

### The affinity of thrombin for ADAMTS-13

As plasma ADAMTS-13 remained unaffected by coagulation, even in the absence of fibrinogen, we explored the effect of ADAMTS-13 concentration upon its initial rate of proteolysis by thrombin. To do this qualitatively, we incubated 5–160 nm ADAMTS-13 with 25 nm thrombin for 60 min, and thereafter assessed proteolysis using the anti-ADAMTS-13 pAb. For each concentration, the same amount of ADAMTS-13 was loaded for blotting. This ensured that the intensity of ADAMTS-13 fragments observed was proportional to their relative rates of proteolysis. ADAMTS-13 cleavage increased with its concentration (Fig. 5E). This observation indicated that the *K*_m_ for this reaction must appreciably exceed the plasma concentration of ADAMTS-13 (5 nm). This contention was further supported when the affinity of ADAMTS-13 for thrombin was measured using an equilibrium plate binding assay (Fig. 5F). We determined a *K*_D_ of ∼95 nm– nearly 20 times greater than the plasma concentration of ADAMTS-13. These data suggest that under normal physiological circumstances, ADAMTS-13 may be protected from proteolysis by the comparatively low affinity of thrombin for the metalloprotease.

## Discussion

ADAMTS-13 is susceptible to proteolytic inactivation by thrombin (and certain other serine proteases) *in vitro* [19,26]. Physiologically, thrombin is generated rapidly, and at high local concentrations during the normal hemostatic response. In these locations, thrombin might readily encounter and so also proteolyze ADAMTS-13 *in vivo*. Consequently, we endeavored to characterize (i) the sites of proteolysis in ADAMTS-13 by thrombin, (ii) how thrombin recognizes ADAMTS-13, (iii) the mode of its inactivation, and (iv) the physiological significance of such proteolysis.

Thrombin proteolyzes ADAMTS-13 at six or seven sites. Of these, the most N-terminal and C-terminal sites are cleaved preferentially [19]. N-terminal sequencing of cleavage fragments demonstrated that the P1 residues of these favored sites correspond to R257 and R1176. This was further confirmed when these residues were mutated to prevent/inhibit thrombin-mediated cleavage. Although we were unable to specifically identify the other, less-favored thrombin cleavage sites, they could be predicted by primary sequence analysis. R287 (at the end of the metalloprotease domain) appears to be susceptible to proteolysis, but this site is cleaved many times slower than R257. The other predicted sites at R393, R415, R910 and R968 are located in positions in accordance with our previous estimates [19]. As these sites were cleaved appreciably slower than those at R257 and R1176, their likely significance with regard to the physiological inhibition of ADAMTS-13 is uncertain. With the exception of the C-terminal CUB domains, all proteolytic fragments remained covalently associated after thrombin cleavage through disulfide bonds.

In order to more accurately establish how thrombin recognizes and cleaves ADAMTS-13, we employed a library of thrombin variants. Previously, we reported the ability of soluble TM (and not heparin) to inhibit proteolysis of ADAMTS-13 by thrombin, suggesting the importance of exosite I (and not exosite II) on thrombin in this reaction [19]. Thrombin interacts with its different substrates and cofactors via these two charged exosites [27]. As only variants carrying substitutions in exosite I (very similar to those required for binding to TM [22]) exhibited impaired ADAMTS-13 cleavage, the functional importance of this region in ADAMTS-13 proteolysis was clearly demonstrated. All exosite II variants tested cleaved ADAMTS-13 normally.

We found that thrombin-cleaved ADAMTS-13 still bound VWF, but with ∼8-fold reduced affinity (*K*_D_∼45 nm). Reduced binding would clearly compromise the ability of ADAMTS-13 to cleave VWF. However, it is likely that there are additional reasons for the inactivation of thrombin-cleaved ADAMTS-13, as reactions containing 10-fold higher concentrations of thrombin-cleaved ADAMTS-13 were devoid of any detectable VWF-cleaving function. Proteolysis of ADAMTS-13 by thrombin after R1176 is predicted to result in the removal of the C-terminal CUB domains from the rest of the molecule. However, the loss of the CUB domains is by itself perhaps unlikely to inactivate ADAMTS-13, as truncated variants lacking these domains have previously been shown to retain VWF-cleaving activity [28–31]. The influence of cleavage after R257 could not be predicted, but naturally because of its location could potentially disrupt the function of the metalloprotease domain.

With a view to measuring the rate at which ADAMTS-13 was inactivated by thrombin, we performed ADAMTS-13 activity assays using VWF115, which enabled kinetic analysis of proteolysis [20]. Intriguingly, we found that thrombin-cleaved ADAMTS-13 remained fully active against this recombinant A2 domain fragment substrate. This observation raises several interesting issues. Firstly, this demonstrates that despite proteolysis at R257, the metalloprotease domain clearly remains functional. Secondly, this highlights that there are fundamental differences between the ADAMTS-13 activity assays that employ multimeric VWF to those that use A2 domain fragments as a substrate. Very possibly, thrombin cleavage ablates a functionality of ADAMTS-13 that is essential for proteolysis of multimeric VWF but that is not required for cleavage of short substrates. Consistent with this was our finding that thrombin-cleaved ADAMTS-13 bound normally to VWF115, but exhibited an 8-fold reduction in affinity for multimeric VWF. It therefore seems likely that there are additional ADAMTS-13-binding sites outside the A2 domain that are important to its ability to proteolyze VWF. In light of these data, analysis of ADAMTS-13 activity in plasma samples using short A2 domain fragments should be carried out cautiously, as results might not necessarily accurately reflect the true VWF-cleaving function of ADAMTS-13 in those instances where ADAMTS-13 proteolysis may have occurred.

Examining the possible proteolysis of ADAMTS-13 by thrombin physiologically during normal hemostasis is problematic. As cleavage might be predicted to occur only locally at sites of vessel injury, this would be unlikely to have a detectable effect upon ADAMTS-13 systemically. Consequently, we were limited to the analysis of ADAMTS-13 proteolysis in plasma *ex vivo*. Previously, we observed ADAMTS-13 proteolysis following initiation of coagulation in plasma, but only when rADAMTS-13 was first added to enable its detection [19]. In the experiments reported here, we could see no clear evidence for fragmentation of endogenous ADAMTS-13. The major reason for this was not because of its protection by VWF, or because of competition with other substrates (such as fibrinogen) for thrombin, but rather because of the comparatively low affinity of thrombin for ADAMTS-13 (*K*_D_∼95 nm). The high *K*_D_ and *K*_m_ for this reaction very possibly protects ADAMTS-13 from rapid proteolysis in plasma, and is consistent with the observed presence of unproteolyzed ADAMTS-13 in serum. That aside, it must be considered that the local concentrations of ADAMTS-13 at sites of vessel damage are likely to be elevated, as VWF is recruited to the injured region. Consequently, whether the plasma experiments monitoring ADAMTS-13 fragmentation are a fair representation of what may happen to ADAMTS-13 physiologically at the site of vessel injury remain uncertain.

Despite our findings, it is clear that ADAMTS-13 is susceptible to proteolysis *in vivo*. This is particularly evident in patients with sepsis-induced disseminated intravascular coagulation (DIC) [26]. It remains unclear, however, which enzyme(s) is responsible for the observed fragmentation of ADAMTS-13. It is conceivable that the elevated and systemic generation of thrombin that occurs during DIC could cause appreciable proteolysis of ADAMTS-13. The proteolytic inactivation of ADAMTS-13 would potentially lead to reduced VWF processing and high concentrations of UL-VWF that could in turn contribute to the pathogenesis of DIC. The identification of the sites of thrombin proteolysis in ADAMTS-13 might be useful in determining whether these same sites are cleaved *in vivo*, and in turn aid in the identification of the enzyme(s) responsible for its physiological fragmentation.
